# The landscape of gene co-expression modules correlating with prognostic genetic abnormalities in AML

**DOI:** 10.1186/s12967-021-02914-2

**Published:** 2021-05-29

**Authors:** Chao Guo, Ya-yue Gao, Qian-qian Ju, Chun-xia Zhang, Ming Gong, Zhen-ling Li

**Affiliations:** Department of Hematology, China–Japan Friendship Hospital, Yinghua East Street, Beijing, China

**Keywords:** Acute myeloid leukemia, Weighted co-expression network analysis, Prognostic signature

## Abstract

**Background:**

The heterogenous cytogenetic and molecular variations were harbored by AML patients, some of which are related with AML pathogenesis and clinical outcomes. We aimed to uncover the intrinsic expression profiles correlating with prognostic genetic abnormalities by WGCNA.

**Methods:**

We downloaded the clinical and expression dataset from BeatAML, TCGA and GEO database. Using R (version 4.0.2) and ‘WGCNA’ package, the co-expression modules correlating with the ELN2017 prognostic markers were identified (R^2^ ≥ 0.4, p < 0.01). ORA detected the enriched pathways for the key co-expression modules. The patients in TCGA cohort were randomly assigned into the training set (50%) and testing set (50%). The LASSO penalized regression analysis was employed to build the prediction model, fitting OS to the expression level of hub genes by ‘glmnet’ package. Then the testing and 2 independent validation sets (GSE12417 and GSE37642) were used to validate the diagnostic utility and accuracy of the model.

**Results:**

A total of 37 gene co-expression modules and 973 hub genes were identified for the BeatAML cohort. We found that 3 modules were significantly correlated with genetic markers (the ‘lightyellow’ module for NPM1 mutation, the ‘saddlebrown’ module for RUNX1 mutation, the ‘lightgreen’ module for TP53 mutation). ORA revealed that the ‘lightyellow’ module was mainly enriched in DNA-binding transcription factor activity and activation of HOX genes. The ‘saddlebrown’ module was enriched in immune response process. And the ‘lightgreen’ module was predominantly enriched in mitosis cell cycle process. The LASSO- regression analysis identified 6 genes (NFKB2, NEK9, HOXA7, APRC5L, FAM30A and LOC105371592) with non-zero coefficients. The risk score generated from the 6-gene model, was associated with ELN2017 risk stratification, relapsed disease, and prior MDS history. The 5-year AUC for the model was 0.822 and 0.824 in the training and testing sets, respectively. Moreover, the diagnostic utility of the model was robust when it was employed in 2 validation sets (5-year AUC 0.743–0.79).

**Conclusions:**

We established the co-expression network signature correlated with the ELN2017 recommended prognostic genetic abnormalities in AML. The 6-gene prediction model for AML survival was developed and validated by multiple datasets.

**Supplementary Information:**

The online version contains supplementary material available at 10.1186/s12967-021-02914-2.

## Background

The prognosis of AML is characterized by clonal cytogenetic and molecular variations harbored by leukemic cells, the prognostic significance of which is validated by previous studies and integrated into the ELN 2017 risk-stratification system [1], which is recommended by the NCCN AML guideline. The prognostic markers include: (1) fusion genes, like RUNX1-RUNX1T1/MLLT3-KMT2A/CBFB-MYH11/DEK-NUP24/BCR-ABL; (2) cytogenetic variants, like − 5 or del(5q), − 17 or abn(17p), − 7; 3) molecular variations, like CEBPA mutation, FLT3-ITD, RUNX1, ASXL1, TP53 etc. The treatment option and prognosis are based on the risk stratification of individual patients. It is reported that specific expression signatures are strongly associating and interacting with some cytogenetic/molecular variation. For instance, the NPM1 mutation was reported to be correlated with over-expression of PBX3 and HOXA gene cluster, which is required for the maintenance of leukemia cells harboring NPM1 mutation [2]. To our knowledge, the association of expression signatures with other important mutations, such as FLT3-ITD/TP53/etc., have not been fully elucidated. Moreover, risk stratification of AML patients is not determined only by signal gene variation, but the combination of multiple gene status. Different mutation status of NPM1 and FLT3-ITD, demonstrate low risk (mutated NPM1 without FLT3-ITD), intermediate risk (mutated NPM1 with FLT3-ITD, or wildtype NPM1 without FLT3-ITD), and adverse risk (wildtype NPM1 with FLT3-ITD). Therefore, this study also aims to uncover the difference of transcriptomic signatures between mutation combinations. The correlation analysis between molecular variant and transcription profiles, will promote the identification of potential valuable prognostic markers and therapeutic targets [3].

Over the recent years, the increasing genomic and expression data has emerged by next generation sequencing, which provided us the chance to identify genetic and transcriptomic markers relating to clinical outcomes. To improve the power of test for correlation analysis, we utilized the BeatAML database, which was the largest RNAseq dataset for AML patients by now [4]. BeatAML database included the clinical/cytogenetic/mutation/expression data originated from 672 patients. The WGCNA was used to identify the co-expression modules based on the scale-free network, and calculate the first principal component of gene modules as module eigengenes (MEs) [5, 6]. Then the target modules were identified, the ME of which was significantly correlated with prognostic markers of ELN2017. The sequential ORA was conducted to reveal the enriched cell signaling pathways for target modules. Furthermore, the LASSO regression analysis was used to reduce the dimensionality and fit survival data to the prediction model based on expression level of hub genes. Due to the batch effect between individual expression datasets, the accuracy of prediction model was limited. However, the successful establishment of the model provided us key genetic variables associating with survival, which will uncover the potential crucial expression signatures.

In this study, we found significantly correlated gene modules to NPM1, RUNX1 and TP53 mutation, the enriched cell signaling pathways of which were identified. The integrated hub genes across modules were input into LASSO analysis and established prediction model of OS, which was validated by 2 external datasets. Our work offered the landscape of expression signatures relating to ELN2017 prognostic markers and revealed the key hub genes and pathways.

## Methods

### Datasets download

The clinical, genetic and transcription matrix was downloaded from BeatAML database [4] (http://www.vizome.org/aml/). 14 available markers were selected for further analysis, including ELN2017 risk stratification, complex karyotype, del(7), RUNX1-RUNX1T1, CBFB-MYH11, biallelic CEBPA mutation, MLL3-KMT2A, DEK-NUP214, GATA2-MECOM, FLT3-ITD, NPM1 mutation, ASXL1 mutation, RUNX1 mutation and TP53 mutation. Then the expression dataset was download in the form of RPKM. After the data was integrated, 421 non-APL AML patients with prognostic markers and RNAseq data were selected for WGCNA. Since the patients in BeatAML accepted various experimental target therapy and non-standard treatment, which lead to unignorable bias for survival analysis. So, we included other expression datasets with survival data to establish and validate the prediction model. We downloaded the OS and expression data from TCGA database (https://portal.gdc.cancer.gov/) (IlluminaHiSeq_RNASeqV2 platform, 136 non-APL AML cases). The microarray data and survival information was obtained from GEO database (https://www.ncbi.nlm.nih.gov/geo/), for GSE12417 [7] (Affymetrix Human Genome U133 Plus 2.0 Array, 79 cytogenetic normal non-APL AML cases) and GSE37642 [8] (Affymetrix Human Genome U133 Plus 2.0 Array, 140 non-APL AML cases). Because no detail allelic ration information was provided by BeatAML database, we followed the recommendation of ELN2017 in such situation, and considered the presence of FLT3-ITD as high risk, unless it co-occurred with NPM1 mutation which was considered as intermediate risk.

All datasets supporting our findings were available from public databases, the last visit was on June 22nd, 2020.

### WGCNA

The whole gene set of RNAseq data were used to construct the co-expression network, by R software (version 4.0.2) and ‘WGCNA’ package [5]. The hierarchical clustering by average link, was implemented to detect outliers. To construct the scale-free network, the minimal beta value setting the scale free R^2^ > 0.85, was defined as soft threshold power. Then the matrix of gene adjacency was generated from the calculation of inter-gene correlation coefficients by Pearson’s method, which was subsequentially turned into the topological overlap matrix (TOM). After the minimal module size was set as 30 genes, the average linkage hierarchical clustering was performed to divide the whole gene set into modules based on TOM-based dissimilarity. Then module membership (the correlation coefficients between individual gene and eigengene in the same module) and gene significance (the correlation coefficients between gene and prognostic markers) were calculated by Pearson’s method. Modules eigengenes (MEs), the first principal component of expression matrix, were correlated to the target prognostic markers. Correlation coefficient R^2^ ≥ 0.4 and p value < 0.01 were set as the criteria for significant correlation between MEs and prognostic markers. Then the hub genes were identified by gene significance ≥ 0.2, module membership ≥ 0.8 and q. Weighted < 0.01 (local FDR adjusted weighted p value of correlation between genes and prognostic markers).

### Protein–protein interaction network of genes in selected modules

STRING (Search Tool for the Retrieval of Interacting Genes/Proteins) database (https://string-db.org/) was used to predict PPI (protein–protein interaction) network information based on the previous evidence and experiments. After mapping the gene symbols into STRING database, minimal criteria for extracting PPI pairs was 0.4. The nodes were calculated and ranked by connectivity degree method, 10 top nodes were screened by cytoscape software (version 3.7.2) and cytohubba plugin.

To demonstrate the biological function and implication on cell signaling of significantly correlated MEs, DAVID [9] (Database for Annotation, Visualization and Integrated Discovery) online tool (https://david.ncifcrf.gov/) was used for gene enrichment analysis based on the Gene ontology (GO) and Kyoto Encyclopedia of Genes and Genomes (KEGG) database. The package ‘ReactomePA’ was implemented for analysis based on Reactome database (https://reactome.org/) [10]. The enriched q value (local FDR adjusted p value) < 0.05 is set as cut-off value for ORA.

### Prediction model for AML survival

The univariate Cox proportional hazard regression analysis was employed to investigate the association of hub genes and OS of AML patients in TCGA AML cohort. Then to minimize the overfitting, we performed the iterative regression analysis (LASSO) to reduce dimensionality of inputted variables, and establish the prediction model of variables with non-zero coefficients. We conducted a bootstrap aggregation approach, and the tenfold cross-validation by ‘glmnet’ package. The TCGA AML cohort was randomly assigned into training (50%) and testing (50%) sets. The risk scores of individual patients were calculated based on the expression level of gene variables with non-zero coefficients. The cutoff value was determined by function ‘surv_cutpoint’ of package ‘survminer’. Kaplan–Meier analysis and time-dependent ROC were performed by ‘survival’ and ‘survivalROC’ packages. Furthermore, the testing set and independent validation cohorts, GSE12417 and GSE37642, were employed to validate the robustness of diagnostic accuracy on overall survival by the prediction model. Then the risk scores of AML patients were compared by unpaired t test, between ELN2017 risk groups, with or without MDS history, newly diagnosed or relapsed patients. Furthermore, the risk scores and other possible prognostic factors, like age, blast percentage, FAB subtypes, type of induction treatment, race, sex, risk stratification of ELN2017 and transplantation, were inputted into multivariate Cox analysis to validate whether the risk score was an independent risk factor for AML survival.

## Results

### Results of WGCNA

**Fig. 1:**
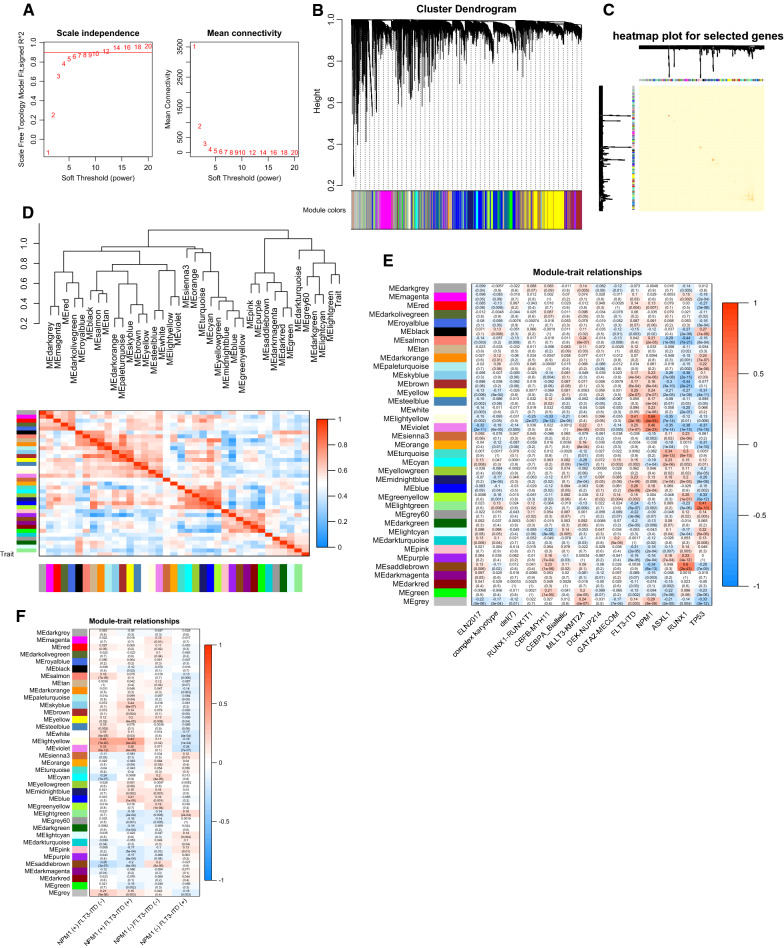
**A** The scale independence (the left plot) and mean connectivity (the right plot) corresponding to different soft-thresholding values. **B** The cluster dendrogram (the upper part) and the co-expression modules (the lower part) generated by average linkage hierarchical clustering method. the branches of the dendrogram represent individual genes. The height indicates the Euclidean distance. Each module that contains weighted co-expressed genes, is displayed with a distinct color. **C** The heatmap of topological overlap using 400 randomly selected genes. The genes are divided into different colors (modules), shown under the cluster dendrogram. **D** The heatmap of module eigengene adjacency, which stands for the relationship between distinct co-expression modules. **E** The module-trait relationship plotter. All modules (colors) are displayed on the longitudinal axis, while all prognostic markers are displayed on the transverse axis. Each cell contains R^2^ and p value of correlations between the modules and prognostic markers by Spearman’s method. The gradient color of each cell corresponds to the R^2^ (red = 1, blue =  − 1). **F** The module-trait relationships for the combos of NPM1 and FLT3-ITD status

The clinical and genetic features of included AML cases in BeatAML and TCGA database were shown in Table 1. The expression data of 421 non-APL AML patients were inputted into WGCNA. No outliers were detected after all samples were hierarchically clustered using average distance and Pearson’s method, the dendrogram for which was shown in Additional file 1: Figure S1. The lowest soft threshold power = 6, by which the scale free R^2^ > 0.85 (Fig. 1A). The calculation based on TOM-based dissimilarity divided the whole gene set into 37 gene modules (Fig. 1B), after we merged the modules with dissimilarity less than 20% by setting the mergeCutHeight as 0.20. 400 randomly selected genes were grouped into modules and generate the heatmap of topological overlap (Fig. 1C), indicating high topological overlap degree of co-expression network in individual modules. Moreover, the correlation of co-expressed modules was demonstrated by module eigengenes adjacency heatmap (Fig. 1D). Finally, the relationship of modules and traits was shown in Fig. 1E. Whereas we noticed that the status of gene mutation combinations will also be of prognostic value rather than the single gene, such as NPM1 and FLT3. Therefore, we performed correlation analysis for modules with different combinations of NPM1/FLT3-ITD mutation status (Fig. 1F). 3 pairs of module-trait were identified to be significant correlation, and were studied sequentially, including ‘lightyellow’ module (R^2^ = 0.41/p = 2e-18 for FLT3-ITD, R^2^ = 0.66/p = 4e-53 for NPM1 mutation), ‘saddlebrown’ module (R^2^ = 0.6/p = 2e-43, for RUNX1 mutation) and ‘lightgreen’ module (R^2^ = 0.41/p = 2e-10, for TP53 mutation). The ‘violet’ module was initially identified as significantly correlated with NPM1 mutation (R^2^ = 0.46/p = 3e-23), while the following detail analysis on NPM1/FLT3-ITD combinations indicated that this module was not significantly related to any combination. So, the ‘violet’ module was excluded from further analysis. According to the criteria of hub genes, 973 hub genes were identified for further analysis (see Additional file 5: Table S1).

**Table 1:** The clinical and genetic features of TCGA and BeatAML cohorts

	BeatAML	TCGA
Patient number	421	136
Female/male	180/241	61/75
Median age (yr)	61	58
Relapsed disease	23(5.46%)	NA
MDS history	41(9.74%)	NA
ELN2017 risk stratification		
Favorable	101(24.0%)	17(12.5%)
Intermediate	156(37.1%)	80(58.8%)
Poor	163(38.7%)	36(26.5%)
Unknown	1(0.2%)	3(2.7%)
ELN2017 prognostic markers		
Complex karyotype	69(16.4%)	18(13.2%)
del(7)	6(1.4%)	NA
RUNX1-RUNX1T1	11(2.6%)	6(4.4%)
CBFB-MYH11	10(2.4%)	8(5.9%)
CEBPA_Biallelic	7(1.7%)	13(9.6%)
MLLT3-KMT2A	13(3.1%)	2(1.5%)
DEK-NUP214	3(0.7%)	NA
GATA2-MECOM	8(1.9%)	NA
FLT3-ITD	95(22.6%)	38(27.9%)
NPM1	108(25.7%)	38(27.9%)
ASXL1	31(7.4%)	2(1.5%)
RUNX1	32(7.6%)	14(10.3%)
TP53	27(6.4%)	11(8.1%)
5- or del(5q)	NA	3(2.2%)
NPM1 (+) FLT3-ITD (−)	59	NA
NPM1 (+) FLT3-ITD ( +)	49	NA
NPM1 (−) FLT3-ITD (−)	100	NA
NPM1 (−) FLT3-ITD ( +)	46	NA

### The ‘lightyellow” co-expressed module

The ‘lightyellow’ module included 143 genes and was positively correlated with FLT3-ITD (R^2^ = 0.41, p = 2e-18) and NPM1 mutation (R^2^ = 0.66, p = 4e-53) respectively. The further analysis on combinations of NPM1 mutation and FLT-ITD, the ‘lightyellow’ module was only found to be related to NPM1 mutation, regardless of the presence of FLT3-ITD. Despite the correlation coefficients didn’t meet the criteria, the potential negative correlations were detected for the ‘lightyellow’ module with ELN2017 risk stratification, complex karyotype, RUNX1-RUNX1T1, CBFB-MYH11, CEBPA biallelic mutation, ASXL1, RUNX1 and TP53 by p value less than 0.01.

**Fig. 2:**
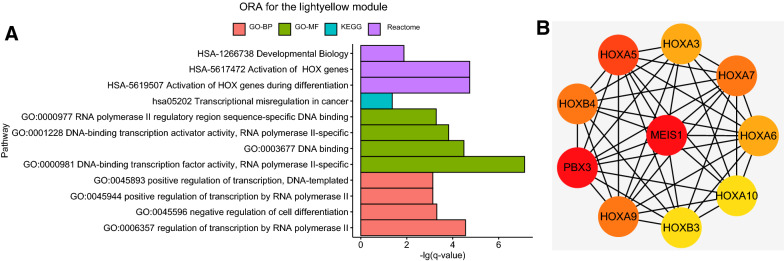
**A** ORA for the lightyellow module (GO/KEGG/Reactome). The y-axis represents the -lg(q value). **B** The top 10 genes with the highest connectivity degrees in PPI network of the ‘lightyellow’ module

The results of ORA for genes in the ‘lightyellow’ module were shown in Fig. 2A. According to GO analysis, the genes were significantly enriched in biological processes like positive regulation of transcription, negative regulation of cell differentiation, etc. And the genes were enriched in molecular functions like RNA polymerase II regulatory region sequence-specific DNA binding, DNA binding, etc. Based on KEGG pathway analysis, the genes were enriched in transcriptional misregulation in cancer. The reactome analysis indicated the genes were enriched in activation of HOX genes, activation of HOX genes during differentiation, etc.

Moreover, the genes of ‘lightyellow’ module were inputted into STRING analysis, resulting in the PPI network shown in Additional file 2: Figure S2. Top 10 genes with highest connectivity degrees among the PPI network were MEIS1, HOXA5, HOXA3, HOXA7, HOXA6, HOXA10, HOXB3, HOXA9, PBX3 and HOXB4 (Fig. 2B).

### The ‘saddlebrown’ co-expressed module

**Fig. 3:**
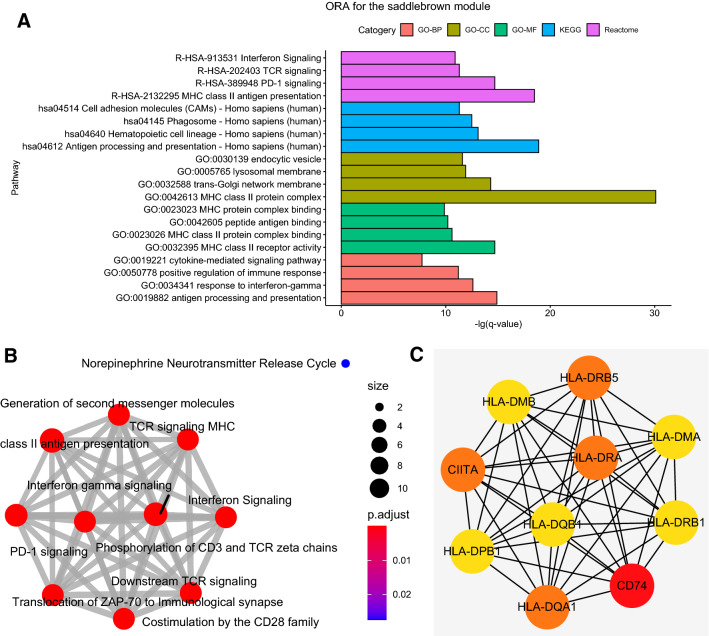
**A** ORA for the saddlebrown module (GO/KEGG/Reactome). The y-axis represents the -lg(q value). **B** The clustering of enriched pathways for the ‘saddlebrown’ module, based on Reactome database. The diameter of dots indicates the count of included genes. The gradient color of dots indicates adjusted p value for enrichment analysis. **C** The top 10 genes with the highest connectivity degrees in PPI network of the ‘saddlebrown’ module

This module included 60 genes and was significantly correlated with RUNX1 mutation (R^2^ = 0.6/ p = 2e-43). The results of ORA for genes in the ‘saddlebrown’ module were shown in Fig. 3A, meanwhile the relationship of enriched Reactome pathways were shown in Fig. 3B. GO analysis revealed that genes were significantly enriched in the following molecular functions: cytokine-mediated signaling pathway, positive regulation of immune response, etc. The biological processes analysis indicated the genes were enriched in MHC protein complex binding, MHC class II protein complex binding, etc. Based on cell component analysis, the genes were enriched in endocytic vesicle, lysosomal membrane, etc. KEGG pathway demonstrated the genes were significantly enriched in hematopoietic cell lineage, antigen processing and presentation, phagosome, etc. According to Reactome analysis, the genes were enriched in interferon signaling, PD-1 signaling, etc.

Furthermore, the genes of ‘saddlebrown’ module were inputted into STRING analysis, resulting in the PPI network shown in Additional file 3: Figure S3. Top 10 genes with highest connectivity degrees among the PPI network (Fig. 3C) included HLA-DRB5, HLA-DMA, HLA-DRB1, CD74, HLA-DQA1, HLA-DPB1, CIITA, HLM-DMB, HLA-DRA and HLA-DQB1.

### The ‘lightgreen’ co-expressed module

**Fig. 4:**
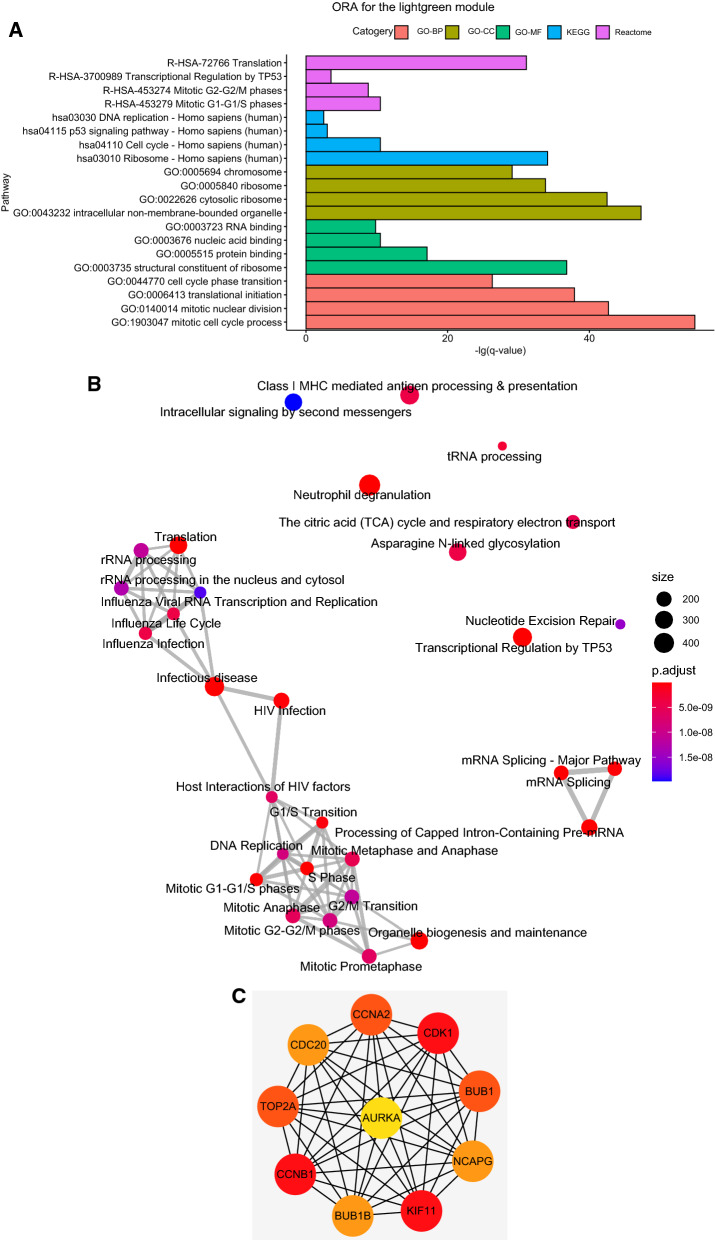
**A** ORA for the lightgreen module (GO/KEGG/Reactome). The y-axis represents the -lg(q value). **B** The clustering of enriched pathways for the ‘lightgreen’ module, based on Reactome database. The diameter of dots indicates the count of included genes. The gradient color of dots indicates adjusted p value for enrichment analysis. **C** The top 10 genes with the highest connectivity degrees in PPI network of the ‘lightgreen’ module

This module included 352 genes and was significantly correlated with TP53 mutation (R^2^ = 0.41/ p = 2e-10). The results of ORA for genes in the ‘lightgreen’ module were shown in Fig. 4A, meanwhile the relationship of enriched Reactome pathways were shown in Fig. 4B. By GO analysis on biological processes, the genes were identified to be enriched in cell cycle phase transition, mitotic nuclear division, etc. Molecular function analysis based on GO database, indicated the genes were enriched in RNA binding, protein binding, etc. And cell component analysis according to GO database, revealed the genes were enriched in chromosome, ribosome, etc. KEGG pathway analysis demonstrated the genes were enriched in DNA replication, p53 signaling pathway, etc. In Reactome analysis, the genes were enriched in Mitotic G2-G2/M phases, transcriptional Regulation by TP53, etc.

Furthermore, the genes of ‘lightgreen’ module were inputted into STRING analysis, resulting in the PPI network shown in Additional file 3: Figure S3. Top 10 genes with highest connectivity degrees among the PPI network (Fig. 4C) included CCNA2, CDK1, BUB1, NCAPG, KIF11, BUB1B, CCNB1, TOP2A, CDC20 and AURKA.

### Results of LASSO penalized regression analysis

The hub genes were reported to have cleaner functional annotations, associate with vital traits (survival time, etc.), and result in better validation [11]. Therefore, hub genes were inputted into LASSO penalized regression analysis, to fit OS of AML patients to the prediction model. After 1000 times of iteration between training and testing set in TCGA AML cohort, an optimized model of 6 gene with non-zero coefficient were identified, including NFKB2, NEK9, HOXA7, APRC5L, FAM30A and LOC105371592. The prediction model for AML OS were established by the 6-gene expression signature, the coefficients of which were listed in Table 2. The risk score for an individual patient was summation of selected gene expression value weighted by coefficients according to Table 2. In detail, risk score = NFKB2* 0.04296 + NEK9* 0.070743 + … + LOC105371592* 0.031033382.

**Table 2:** The prediction model for AML OS. The risk score of individual patients equals to the summation of products of included gene expression level and the corresponding coefficient

ENSEMBL ID	Gene symbol	Gene name	Coefficent
ENSG00000077150	NFKB2	Nuclear factor kappa B subunit 2	0.04296
ENSG00000119638	NEK9	NIMA related kinase 9	0.070743
ENSG00000122592	HOXA7	Homeobox A7	0.055637
ENSG00000136950	ARPC5L	Actin related protein 2/3 complex subunit 5 like	0.748162
ENSG00000226777	FAM30A	Family with sequence similarity 30 member A	0.294823
ENSG00000262050	LOC105371592	Uncharacterized LOC105371592	0.031033

### The association of 6-gene expression signature with traditional risk factors of AML

**Fig. 5:**
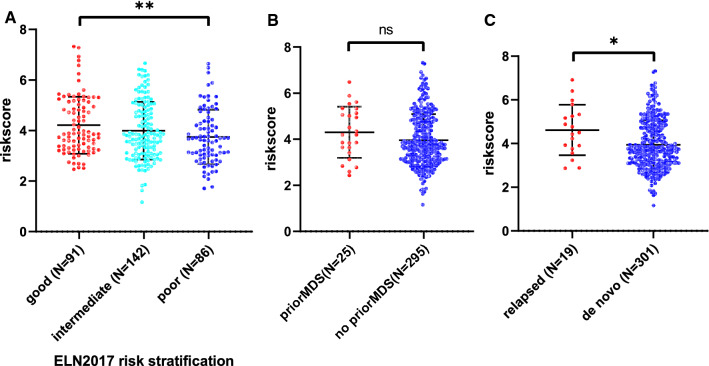
The comparison of risk scores in different ELN2017 risk groups (**A**), with or without MDS history (**B**), de novo or relapsed AML (**C**). **p value < 0.01; *p value < 0.05

The risk scores were calculated for 6-gene expression signature for the individual patients in TCGA AML cohort. The risk scores of patients in the adverse ELN2017 risk group, were significantly higher than that of favorable group (t = 2.799, df = 175, p = 0.0057, Fig. 5A). Meanwhile, AML patients with MDS history, had insignificantly higher risk scores than that without MDS history (t = 1.473, df = 318, p = 0.1418, Fig. 5B). The relapsed AML patients had higher risk scores than de novo AML patients (t = 2.556, df = 318, p = 0.0110, Fig. 5C).

### The validation of 6-gene signature model

**Fig. 6:**
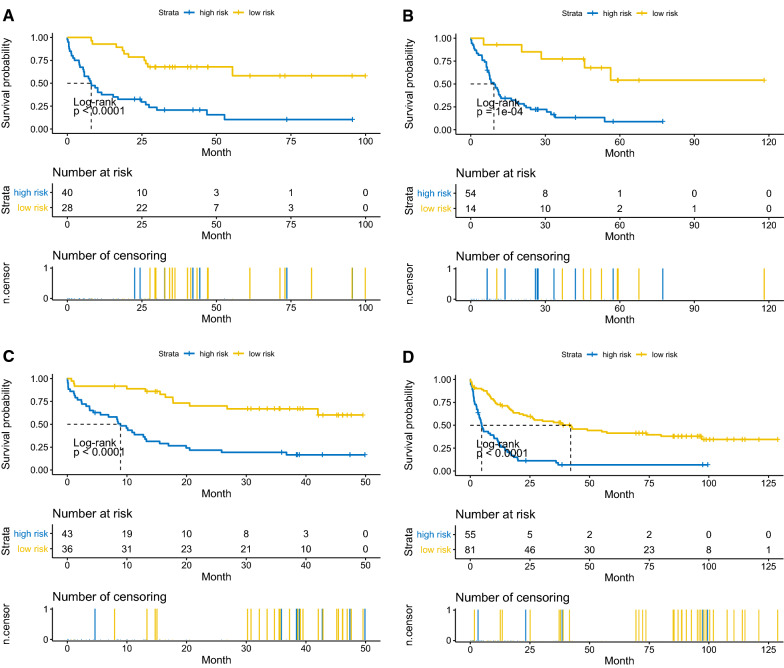
Overall survival analysis based on the 6-gene signature by Kaplan–Meier plotter, for the training set (**A**), testing set (**B**), GSE12417 (**C**) and GSE37642 (**D**)

**Fig. 7:**
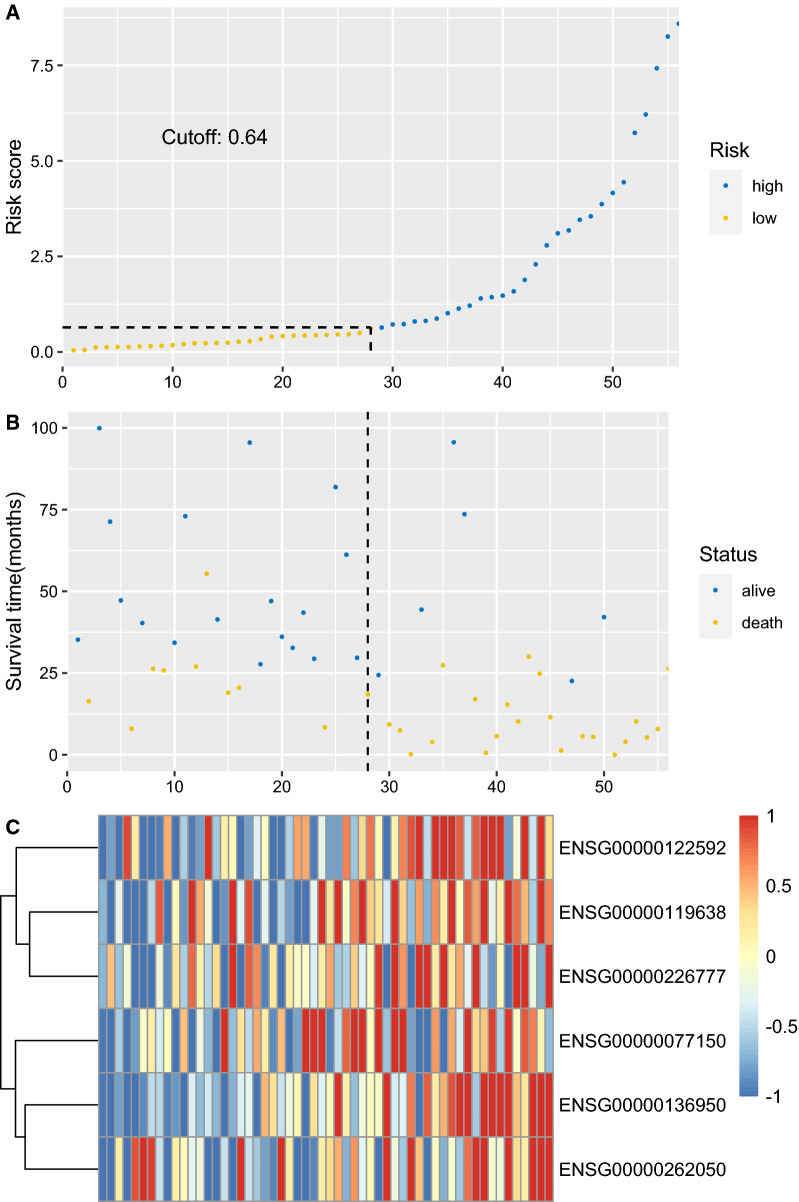
**A** The distribution and cut-off value of 6-gene risk scores in the training set. The cut-off value is 0.64 for risk scores. **B** The survival time and status of the training set corresponding to risk scores. The left half, separated by a line of dashes, included low-risk group, while the right part included high-risk group. The cut-off value is 0.64 for risk scores. **C** The heatmap and hierarchical clustering of the 6-gene model for the training set of TCGA cohort. The ENSEMBL id of genes are displayed on the right longitudinal axis; the clustering dendrogram of genes are displayed on the left longitudinal axis. The relative expression level of genes is indicated by gradient color from blue (− 1) to red (1)

**Fig. 8:**
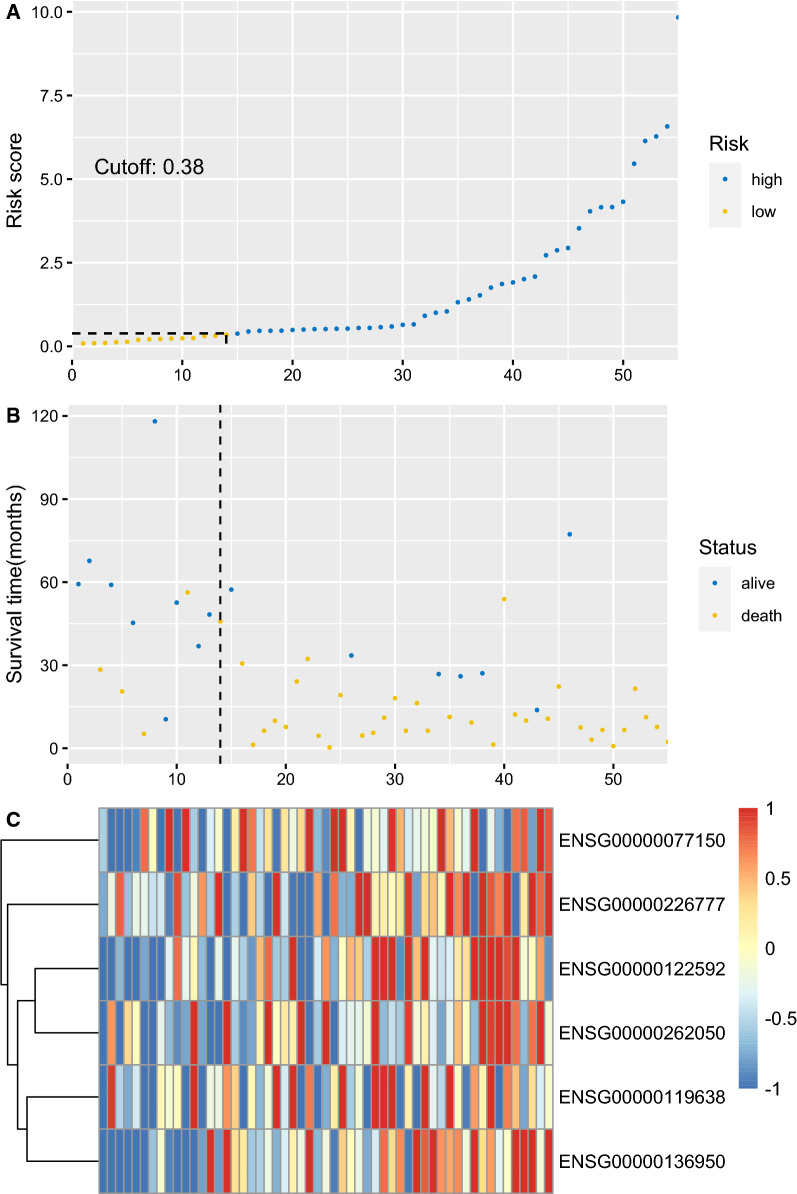
**A** The distribution and cut-off value of 6-gene risk scores in the testing set of TCGA cohort. The cut-off value is 0.38 for risk scores. **B** The survival time and status of the testing set corresponding to risk scores. The left half, separated by a line of dashes, included low-risk group, while the right part included high-risk group. The cut-off value is 0.38 for risk scores. **C** The heatmap and hierarchical clustering of the 6-gene model for the testing set. The ENSEMBL id of genes are displayed on the right longitudinal axis; the clustering dendrogram of genes are displayed on the left longitudinal axis. The relative expression level of genes is indicated by gradient color from blue (− 1) to red (1)

**Fig. 9:**
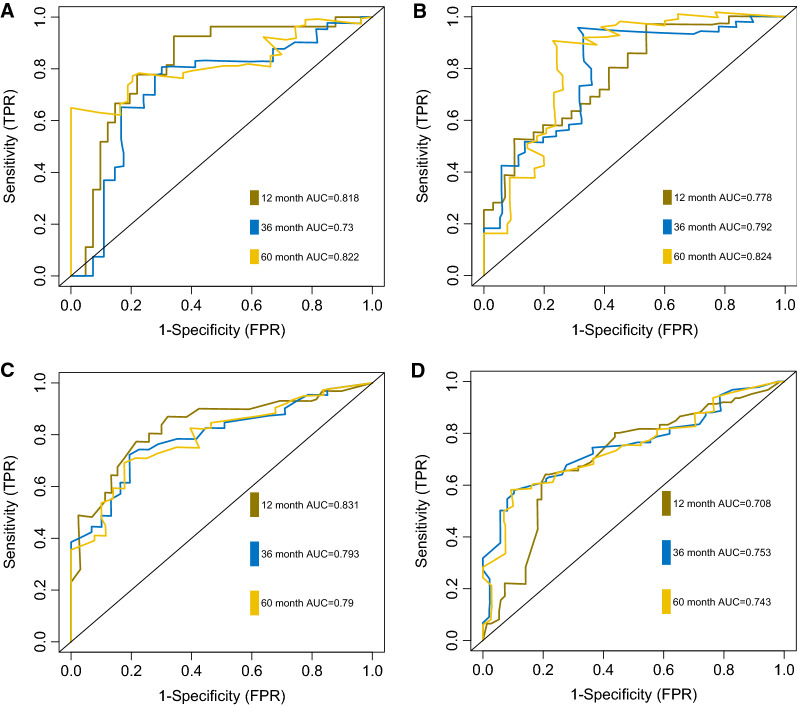
The time-dependent ROC curves showing diagnostic utility of 6-gene signature by AUC, for the training set (**A**), the testing set (**B**), GSE12417 (**C**) and GSE37642 (**D**)

The diagnostic utility was evaluated in the training set, testing set and 2 external independent validation sets. After the cut-off values for risk scores were calculated in each cohort and divided patients into low and high risk groups, the Kaplan–Meier plots were used to compare the OS between groups by log-rank test. The OS in low risk group was significantly longer than that in high risk group in all 4 sets (Fig. 6A–D). In the TCGA training set, median OS was not reached in low risk group, and 8.08 months in high risk group (HR = 4.203, 95%CI 2.251–7.847, p < 0.0001). The median OS was also not reached in low risk group, and 9.30 months for high risk group (HR = 3.342, 95%CI 1.817–6.145, p = 1e-4). Similar results were uncovered in GSE12417 (HR = 3.188, 95%CI 1.720–5.910, p < 0.0001) and GSE37642 (HR = 2.489, 95%CI 1.658–3.737, p < 0.0001). The distribution of risk scores, survival time and gene-survival heatmap were shown for the training set (Fig. 7) and the testing set (Fig. 8) of TCGA cohort. As AUC is a very crucial indicator for utility of a prognostic model, the time dependent ROC analysis was performed for the 4 set (Fig. 9A–D). The 5-year AUC for the training and testing set were 0.822 and 0.824 respectively, whereas it is 0.79 and 0.743 in GSE12417 and GSE37642, respectively, which demonstrated the superiority and robustness in expression datasets generated from different platforms. The results of multivariate COX regression analysis were shown in Table 3, indicating the risk factor was an independent risk factor for OS of AML patients.

**Table 3:** The results of univariate and multivariate Cox analysis of traditional prognostic factors and risk scores generated by the prediction model

	Univariate Cox analysis			Multivariate Cox analysis		
Characteristics	Hazard Ratio	95% CI	p value	Hazard Ratio	95% CI	p value
Age	1.03	1.02–1.05	0	1.01	0.99–1.03	0.298
Blast_percentage	1	0.99–1.02	0.443	NA	NA	NA
FAB	1.06	0.94–1.2	0.351	NA	NA	NA
Induction	3.51	2.19–5.61	0	1.84	0.98–3.46	0.057
Race	0.86	0.66–1.13	0.287	NA	NA	NA
Risk_cytogenetic	1.73	1.23–2.41	0.001	0.64	0.22–1.92	0.43
Risk_molecular	1.83	1.31–2.56	0	3.36	1.2–9.44	0.021
Riskscore	1.01	1–1.02	0.012	1.01	1–1.02	0.023
Sex	1.05	0.69–1.57	0.831	NA	NA	NA
Transplantation	0.41	0.27–0.62	0	0.42	0.25–0.72	0.002

## Discussion

The heterogeneity of expression profiles has been studied for AML patients harboring different mutations or chromosomal abnormality [12–15], but the co-expressed gene modules have not been rarely linked to the genetic markers using WGCNA. Ravasz et al. demonstrated hierarchical network modularity for metabolic network, based on which they proposed the utility of Topological Overlap Matrix to measure how strongly the nodes are connected [16]. Then the concept was transplanted to gene expression network to investigate the scale-free properties by developing a clustering method (WGCNA), by which the gene modules were identified, with high co-expression and strong network connectivity [6].

In the present study, the results of WGCNA indicated that the ‘lightyellow’, ‘saddlebrown’ and ‘lightgreen’ were significantly correlated with NPM1, RUNX1 and TP53 mutation, respectively. In Fig. 1E, the ‘lightyellow’ module was insignificantly or negatively correlated with all genetic variations, except NPM1 and FLT3-ITD. Then the correlation analysis with NPM1/FLT3 combinations indicated that ‘lightyellow’ module was the NPM1-specific co-expression module. ORA indicated that genes in this module were mainly enriched in regulation of transcription and cell differentiation, the molecular function of which involved in RNA polymerase II-specific DNA binding transcription factor activity. In the ‘lightyellow’ module, the HOXA family genes (HOXA1-7/HOXA9-11/HOXA13) and HOXB family genes (HOXB2-9), and the cofactors of HOXA (PBX3/MEIS1) were involved in the abovementioned pathways [17, 18]. The HOX9-11, PBX3 and MEIS1 were also involved in the ‘Transcriptional misregulation in cancer’ pathway (KEGG database), and ‘Activation of HOX genes during differentiation’ pathway (Reactome database). The sequential PPI network analysis also supported the central position of HOXA/B family genes and their cofactors, which demonstrated they have the highest connectivity degrees among the ‘lightyellow’ module. The deregulation of HOX genes and their cofactors was initially reported in AML patients harboring MLL fusion genes [19–21], and played a role in leukemogenesis in this type of AML [22]. The similar expression signature was revealed in NPM1 mutated AML, in which upregulation of PBX3 and HOX9 was required to maintain the survival of leukemic cells [2, 23], and was related to unfavorable clinical outcomes [12, 24]. Additionally, similar WGCNA was performed using TCGA AML data, the results of correlation analysis between co-expression modules and genetic variants were shown in Additional file 4: Figure S4. The BeatAML dataset (including 421 non-APL AML cases) have more samples than that of TCGA dataset (including 136 non-APL AML cases). The statistic power was improved when more samples were inputted into analysis, which decreased the probability of committing type II errors (false negative). Therefore, the analysis based on BeatAML dataset have higher probability of detecting the significant correlation between gene modules and genetic variations, than that based on TCGA dataset. Notably, we noticed there was also a strongly significantly correlated module with NPM1 mutation (R^2^ = 0.71/ p = 4e-28), 45 out 109 genes (Additional file 6: Table S2) in which were overlapped with the ‘lightyellow’ module, including HOXA/B gene clusters and PBX3/MEIS1. These results from clinical and experimental studies confirmed the accuracy of our module analysis and identification of key genes. Considering NPM1 mutation is the most common genetic variant in AML, the specific expression signature characterized by HOXA/B-PBX3-MEIS1, will provided insights to further studies.

For RUNX1 mutation, the ‘saddlebrown’ module was the most correlated co-expression module (Fig. 1E). Corresponding to the mutant exclusivity of NPM1 and RUNX1 mutations reported previously [25], the ‘saddlebrown’ module seems to be underexpressed in patients harboring NPM1 mutation (R^2^ =  − 0.34/ p = 6e-13). The results of ORA indicated the genes of ‘saddlebrown’ were mainly enriched in pathways involving in immune response, cytokine signaling and antigen presentation. These genes are also implicated in the enriched KEGG pathways, including antigen processing and presentation, hematopoietic cell linage, phagosome, and cell adhesion (Fig. 3A). Figure 3B showed the interaction network of ‘saddlebrown’ module enriched pathways by Reactome database, which predominantly composed of immune signaling. The core part with highest connectivity degrees in the ‘saddlebrown’ module, included HLA gene cluster, CD74 and CIITA (Fig. 3C). CD74 is known as the chaperone for MHC class II molecules implicating in antigen presentation [26], the role of which in AML remained unclear. While a recent single cell transcriptomic analysis demonstrated CD74 was expressed at high level in AML cells instead of normal myeloid cells [27]. The prognostic value of CD74 and association with RUNX1 mutation still need further study to validate. CIITA is a vital regulator of MHC class II gene expression including HLA-DR, HLA-DP and HLA-DQ, deregulation of which promoted abolishment of recognition from donor T cell, and lead to AML relapse after allo-HCT [28]. The dysregulation of immune signaling pathways and MHC class II regulator (CD74 and CIITA) may contribute to the inferior clinical outcomes in AML patients harboring RUNX1 mutation.

Since TP53 encodes a DNA-binding transcription factor inducing cell growth arrest and apoptosis upon various cellular stress [29]. Missense or null mutations of TP53 is one of the most powerful independent markers for adverse prognosis in AML [30, 31]. As the results of ORA, the upregulation of mitotic process related pathways in AML patients harboring TP53 mutation, probably resulted from abolishment of TP53 induced cell cycle arrest and apoptosis, which may promote the leukemic cell proliferation and disease progression. Therefore, the regulation of mitotic exit, which refers to the transition from mitosis to interphase, is crucial for TP53 mutated AML. Liu et al. divided the regulator of mitosis exit into 4 groups: (1) APC/C; (2) cyclin B; (3) mitosis kinase and phosphatase; (4) kinesin and microtube-binding proteins [32], all of which were partly overlapped with genes in the ‘lightgreen’ model. Among the top genes in the module, CDC20, encoding cell division cycle protein 20 homolog, is required for anaphase promoting complex/cyclostome (APC/C) to confer full ubiquitin ligase activity and substate specificity. The aberrant expression of CDC20 was reported in AML and positively correlated with EZH2 and TET2 in AML [33], suggesting epigenetic factors participated in the regulation of CDC20. BI-D1870, the inhibitor of RSK, potentiated anti-leukemia activity of vincristine, which prevent the association of activator CDC20 with APC/C and impeded mitosis exit [34]. Other preclinical studies were performed to explore the anti-tumor mechanism of APC/C inhibitors, in cervical cancer, osteosarcoma, colorectal and lung cancer [35, 36]. The association of CDC20 upregulation with TP53 null mutation or functional silencing was uncovered in aneuploid AML [37], which suggested CDC20 as a potential target and biomarker in TP53 mutant AML. The regulatory subunit of cyclin B (encoded by CCNB1) and CDK1 (encoded by CDK1) constitutes the mitosis promoting factor (MPF), which controls the entry or exit of mitosis [38]. Overexpression of CCNB1 was associated with various cancer types [39–42], and predicted inferior response to mTOR inhibitor [43]. The direct association of CCNB1/CDK1 with TP53 has not been validated in AML. 3 main mitotic kinases included Aurora kinases, PLK1 and PP2A. AURKA was one of core genes in PPI network regarding TP53 mutation (Fig. 4C). Aurora Kinase A, encoded by AURKA, is implicated in cell mitosis process, including centrosome maturation and spindle formation [44, 45]. The anti-leukemic effect of Aurora kinase inhibitors was reported via inducing mitochondrial impairment [46], cell cycle arrest[47] etc. Preliminary clinical studies have been conducted to validate the efficacy and safety of Aurora kinase inhibitors in AML [48–50]. Although the association of TP53 mutation and AURKA expression has been rarely studied previously for AML, it was explored in solid tumors with high frequency of TP53 variants, including adrenocortical carcinoma [51], pancreatic cancer [52], head and neck cancer [53], hepatocellular carcinoma [54] and ovarian cancer [55]. Moreover, in cancer cell lines lacking p53, resulting from genetic engineering to express HPV16-E6 oncoprotein or siRNA targeting TP53, the inhibitor of Aurora kinases (VX680) induced apoptosis was enhanced in comparison with cell lines with wide-type p53 [56]. PLK1 was also in the ‘lightgreen’ module, which encoded Polo-like kinase 1. PLK1 is a crucial regulator of multiple processes [57], including mitotic spindle assembly, chromatid separation, activation of Cyclin B/CDK1 complex [58], etc. PLK1 was found to be overexpressed in various AML cell lines and the majority of AML patients [59]. PLK1 inhibitor or siRNA targeting PLK1 blocked the proliferation of AML cell lines, while the normal hematopoietic progenitors were less sensitive to abolishment of PLK1 [60]. This result provided the rationale for targeting PLK1 in the treatment of AML, the clinical trials had been conducted [61, 62]. The association of PLK1 over-expression and silencing of TP53 was reported in aneuploid AML [37]. Kinesin superfamily proteins (KIFs) function mainly as molecular motors binding to and moving across the microtube network [63, 64]. Seven members of KIF family (KIF11, KIF14, KIF15, KIF18A, KIF18B, KIF20A and KIF23) were included in the ‘lightgreen’ module. KIF 11 and KIF23 are the well-studies KIF family members and considered as an oncogene, inhibition of which can cause arrest at mitosis exit in hepatocellular cell carcinoma, lung cancer, pleural mesothelioma, and glioma cancer, breast cancer, meningiomas [65–70]. A phase I clinical trial on the highly selective kinesin spindle protein inhibitor, ARRY-520, has been conducted on advanced AML patients [71]. As the expression level of mitosis exit regulators was positively correlating with TP53 mutation, targeting at these regulators (APC/C, Aurora kinases, PLK1, KIFs) seems to be a reasonable strategy for TP53 mutant AML patients.

No significant correlating modules were detected for other genetic markers in ELN2017, including complex karyotype, del(7), RUNX1-RUNX1T1, CBFB-MYH11, CEBPA biallelic mutation, MLLT3-KMT2A, DEK-NUP214, GATA2-MECOM and ASXL1 mutation. This may partly be attributed to insufficient samples. A quantity of gene variations for one specific abnormality (complex karyotype, del(7), ASXL1) may also abolish the accuracy of our analysis.

The 6-gene signature with non-zero coefficients was identified by LASSO penalized regression analysis. AML patients with traditional risk factors (ELN2017 adverse risk stratification, with MDS history, relapsed disease) have higher risk scores of 6-gene signature (Fig. 5A–C). The Kaplan–Meier analysis demonstrated much better survival profiles of low risk group than that of high risk group for the training set, the testing set and 2 validation sets. Moreover, the multiple Cox proportional hazards regression analysis indicated that 6-gene risk scores were an independent risk factor for AML survival (Table 3). The time dependent ROC was employed to illustrate the diagnostic utility of the 6-gene model, which demonstrated the superior 5-year AUC in the training and testing sets (0.822 and 0.824, respectively). To evaluate the robustness of this model across different datasets, GSE12417 and GSE37642 were used to fit the OS data into 6-gene model. Owing to the different methods of measuring expression level in training/testing sets (RNAseq) and validation sets (microarray), a slight lower 5-year AUCs were estimated for the 2 validation sets (0.79 and 0.743), suggesting that this model was robust across different datasets. NFKB2, encoding nuclear factor NF-kappa-B p100 subunit, was in the ‘brown’ module, which was correlated with FLT3-ITD with a significant p value (6e-4, Fig. 1E). Through a non-canonical pathway, the phosphorylation of NFKB2/p100 leads to its proteolytic process and formation of NF-kappa-B RelB-p52 complex [72]. The study using MV-4–11 cell line, demonstrated FLT3-ITD promoted activation of NF-kappa-B RelB-p52 complex and repressed the expression of DAKP1 in association with HDACs [73]. Since the DAKP1 was acknowledged as tumor suppressor via inducing apoptosis and autophagy, and the abolishment of DAKP1 expression occurred in various cancers, including AML [74]. Therefore, the relationship between NFKB2 expression and FLT3-ITD was intriguing and provided potential biomarkers for HDAC inhibitors in FLT3-ITD AML. NEK9 encodes serine/threonine-protein kinase Nek9, which played a crucial role in G1/S phase transition and S phase progression [75]. Few studies focused on the association of NEK9 and AML, while Matthew et al. identified NEK9 with increased activity or abundance in the imatinib resistant cell model of CML [76]. HOXA7 is a member of HOXA family, which is one of the most studied gene clusters in AML. HOXA7 is identified as potential prognostic markers in AML previously [77]. The association of ARPC5L, FAM30A and LOC105371592 with AML survival or pathogenesis, has not been described previously. Our LASSO analysis provided a robust prediction model for AML survival, and several potential biomarkers or therapeutic targets.

## Conclusion

We identified the significantly correlated gene co-expression modules with prognostic cytogenetic or molecular markers of ELN2017. The sequential ORA illustrated the involved pathways for the key modules. Additionally, the novel prediction model was established with robust and superior diagnostic utility based on hub genes obtained from WGCNA.

## Supplementary Information

**Additional file 1:** **Figure S1.** The hierarchical cluster dendrogram of BeatAML samples. The tips of dendrogram referred to sample ID in BeatAML database. No obvious outliers were found in this figure.

**Additional file 2: Figure S2.** The whole PPI network for the ‘lightyellow’ module.

**Additional file 3: Figure S3.** The whole PPI network for the ‘saddlebrown’ module.

**Additional file 4: Figure S4.** The results of module-trait relationship by WGCNA using TCGA database.

**Additional file 5: Table S1.** The hub genes obtained from WGCNA for AML.

**Additional file 6: Table S2.** The overlapped genes of modules correlating with NPM1 significantly, by analysis based on TCGA and BeatAML database, respectively.

## Data Availability

The datasets analyzed during the current study are available in the GEO database (https://www.ncbi.nlm.nih.gov/gds/), TCGA database (https://portal.gdc.cancer.gov/) and BeatAML database (http://www.vizome.org/aml).

## Abbreviations

AML: Acute myeloid leukemia
ELN: European LeukemiaNet
WGCNA: Weighted gene correlation network analysis
RPKM: Reads per kilobase per million mapped reads
ORA: Over-representation analysis
LASSO: Least absolute shrinkage and selection operator
TCGA: The cancer genome atlas
OS: Overall survival
AUC: Area under curve
RO: Receiver operating characteristic curve
GE: Gene Expression Omnibus
PP: Protein–protein interaction
