# Supplementary figures and images for: The landscape of gene co-expression modules correlating with prognostic genetic abnormalities in AML
Source: J Transl Med. 2021 May 29;19:228. doi: 10.1186/s12967-021-02914-2 (PMC8164775; doi:10.1186/s12967-021-02914-2)

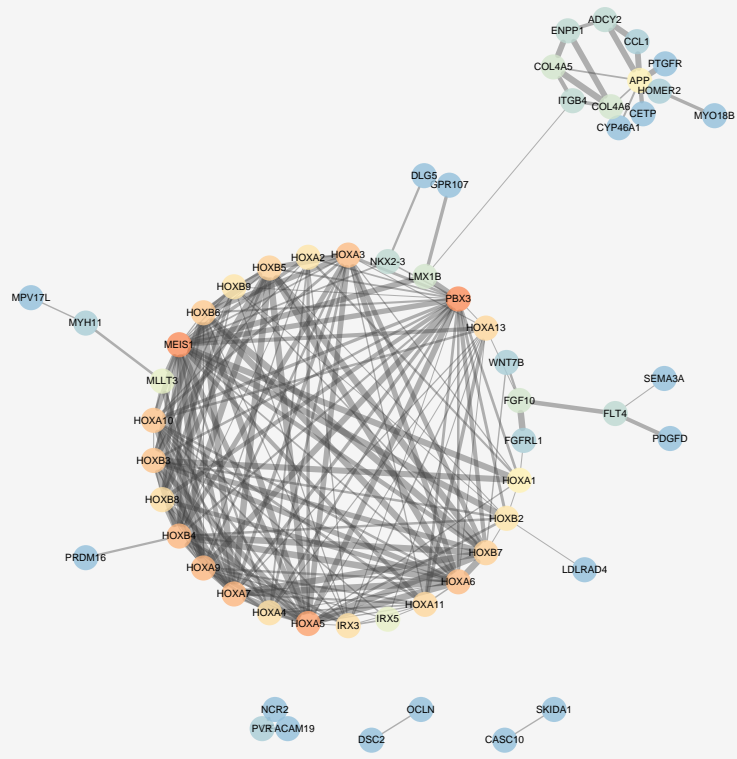

Supplement: Supplementary file 2 — Additional file 2: Figure S2. The whole PPI network for the ‘lightyellow’ module. [file 12967_2021_2914_MOESM2_ESM.pdf]

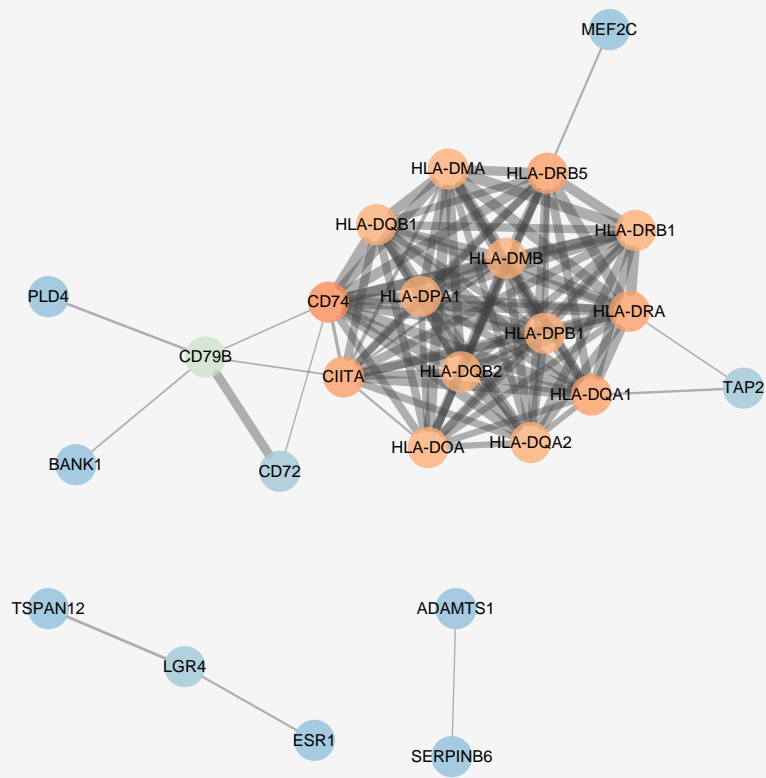

Supplement: Supplementary file 3 — Additional file 3: Figure S3. The whole PPI network for the ‘saddlebrown’ module. [file 12967_2021_2914_MOESM3_ESM.pdf]

Module-trait relationships

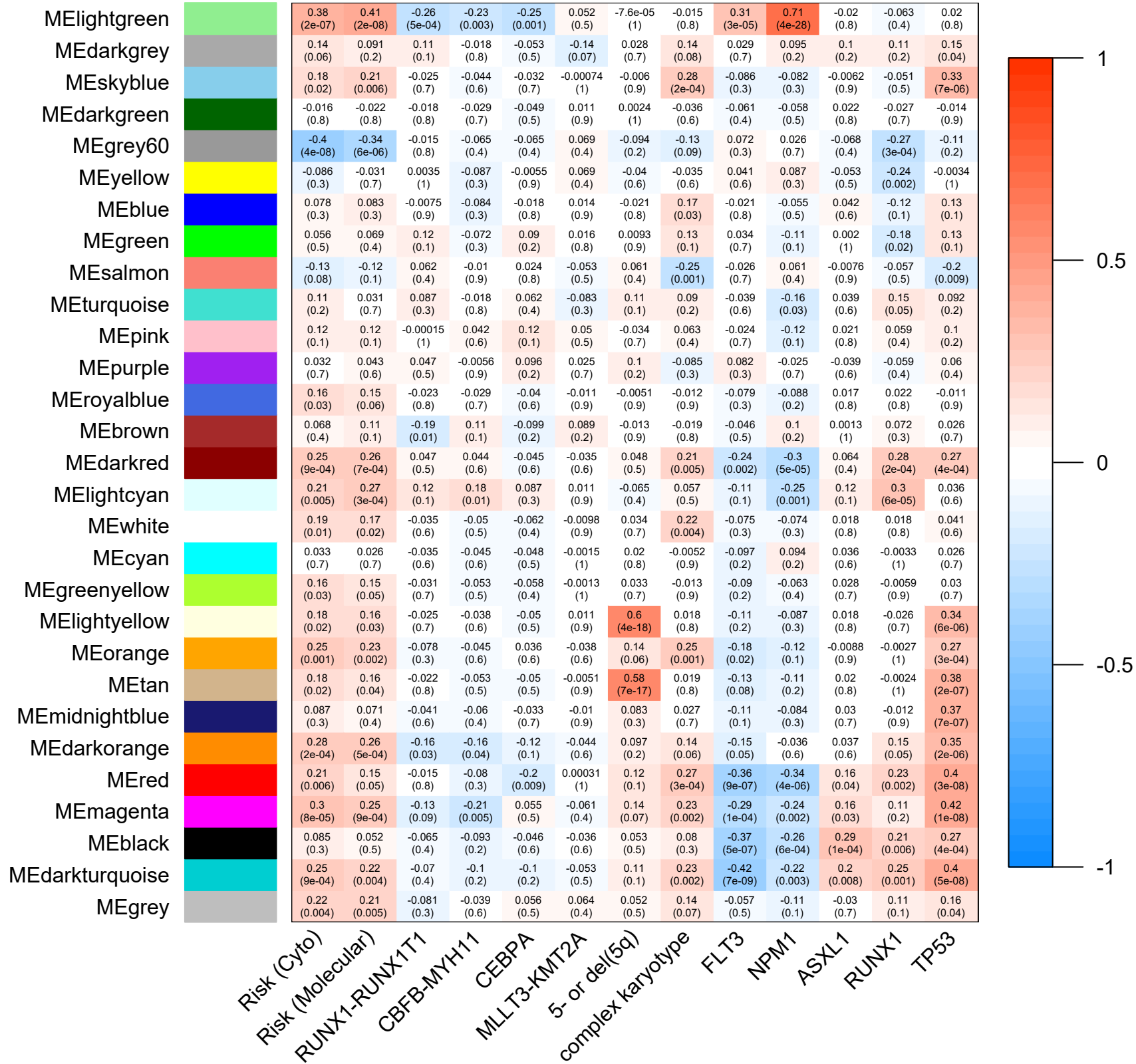

Supplement: Supplementary file 4 — Additional file 4: Figure S4. The results of module-trait relationship by WGCNA using TCGA database. [file 12967_2021_2914_MOESM4_ESM.pdf]
