# Supplementary material for: The landscape of gene co-expression modules correlating with prognostic genetic abnormalities in AML
Source: J Transl Med. 2021 May 29;19:228. doi: 10.1186/s12967-021-02914-2 (PMC8164775; doi:10.1186/s12967-021-02914-2)
Supplement: Supplementary file 1 — Additional file 1: Figure S1. The hierarchical cluster dendrogram of BeatAML samples. The tips of dendrogram referred to sample ID in BeatAML database. No obvious outliers were found in this figure. [file 12967_2021_2914_MOESM1_ESM.pdf]

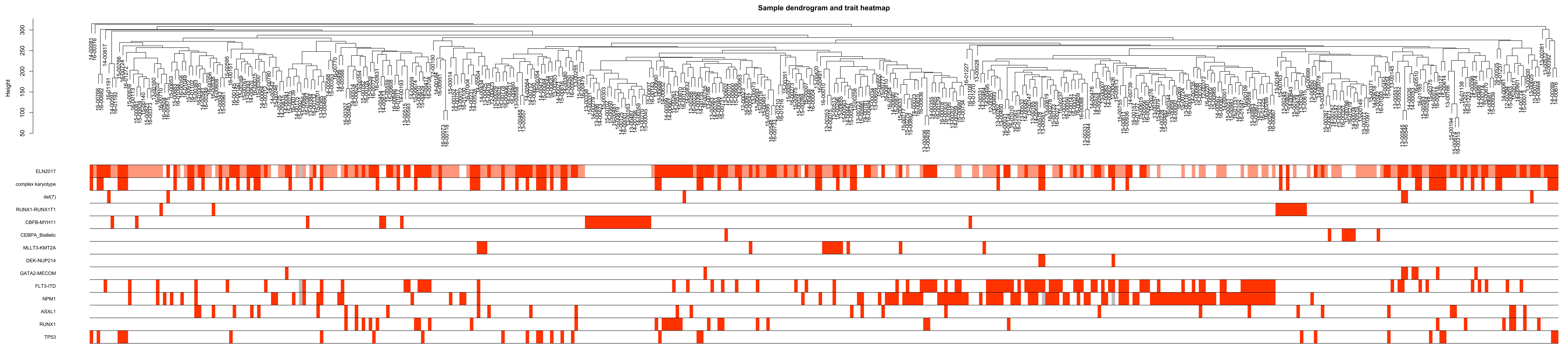
Sample dendrogram and trait heatmap
Height
300
250
200
150
100
50
ELN2017
complex karyotype
del(7)
RUNX1-RUNX1T1
CBFB-MYH11
CEBPA_Biallelic
MLLT3-KMT2A
DEK-NUP214
GATA2-MECOM
FLT3-ITD
NPM1
ASXL1
RUNX1
TP53
